# Inference of Gene Regulatory Networks Using Bayesian Nonparametric Regression and Topology Information

**DOI:** 10.1155/2017/8307530

**Published:** 2017-01-04

**Authors:** Yue Fan, Xiao Wang, Qinke Peng

**Affiliations:** Systems Engineering Institute, School of Electronic and Information Engineering, Xi'an Jiaotong University, Xi'an 710049, China

## Abstract

Gene regulatory networks (GRNs) play an important role in cellular systems and are important for understanding biological processes. Many algorithms have been developed to infer the GRNs. However, most algorithms only pay attention to the gene expression data but do not consider the topology information in their inference process, while incorporating this information can partially compensate for the lack of reliable expression data. Here we develop a Bayesian group lasso with spike and slab priors to perform gene selection and estimation for nonparametric models. B-spline basis functions are used to capture the nonlinear relationships flexibly and penalties are used to avoid overfitting. Further, we incorporate the topology information into the Bayesian method as a prior. We present the application of our method on DREAM3 and DREAM4 datasets and two real biological datasets. The results show that our method performs better than existing methods and the topology information prior can improve the result.

## 1. Introduction

Gene regulatory network plays an important role in diverse cellular functions. A reliable method to identify the structure and dynamics of such regulation is important for understanding complex biological processes and is helpful for treatment of diseases. With the development of high throughout technologies in recent years, gene expression data has provided a useful way to investigate the cellular system.

Generally, there are two types of gene expression data used to predict the structure of GRNs, which are steady-state data and time-series data. The steady-state data measures the steady-state levels in different samples, while time-series data measures the expression levels at several successive time points. Since the time-series data contains the dynamic information of the network while the steady-state data does not [1], we focus on the time-series data in this paper.

Over the last several years, a number of network inference methods have been developed to tackle this problem, including Bayesian network [2, 3], dynamic Bayesian network [4, 5], Boolean network [6, 7], ordinary differential equation [8, 9], and mutual information [10, 11]. A comprehensive review can be found in [12, 13]. Among these methods, dynamic Bayesian network has become the major focus for inferring gene regulatory network because it can infer causal interactions, model cyclic interactions, and has less computational complexity than ordinary differential equation.

Inferring a GRN from time-series data is known to be challenging partly due to the high number of genes relative to the number of data points. More importantly, the interactions between genes are typically nonlinear; thus linear model may be inefficient to recognize the nonlinear interactions. A flexible way to solve this problem is to use B-spline functions to describe the nonlinear interactions, and the B-spline functions have been used to infer GRNs in previous studies [14, 15]. A key problem in spline regression is the knot selection which greatly influences the curve fitting. Reference [14] suggested using penalized-splines to avoid overfitting and reduce the number of parameters to be estimated. Among many penalized methods, lasso [16] is the most popular method due to its ability to select and estimate simultaneously and can produce exact 0 estimates. Group lasso [17] was also developed to select grouped variables. Reference [18] proposed group lasso or Bayesian group lasso when spline regression was used because the predictors belong to a same gene forming a natural group. Reference [19] also developed a Bayesian adaptive group lasso to perform simultaneous model selection and estimation for B-spline regression. However, Bayesian spline regression methods still predict a lot of false positive interactions because of the indirect effects existing in the GRNs.

Recently, [20] proposed a new method which uses network topology information to improve gene regulatory network inference; they used a prior that both prokaryotic and eukaryotic transcription networks exhibit an approximately scale-free out-degree distribution while the in-degree distribution is a more restricted exponential function; this structure property is described in [21]. Reference [20] also pointed out that 79% or more genes regulators are less than 3. This property means that most genes in a GRN are regulated by a few regulators and may be possible to be combined with the B-spline regression to improve the results of the GRN inference.

In this paper, we work with a dynamic Bayesian network and use spline regression to detect the nonlinear interactions between genes. A Bayesian group lasso is also used to avoid overfitting and reduce the number of parameters to be estimated. Comparing with group lasso, Bayesian group lasso is a better choice because there are 2 major advantages of Bayesian selection methods: (1) The tuning parameter can be set flexibly. (2) The topology information can be incorporated easily. Further, instead of taking a traditional Bayesian group lasso, we use a Bayesian group lasso model with spike and slab priors since this problem only requires the sparsity on the group level and spike and slab priors can exclude or include the entire group of B-spline basis functions. Finally, we incorporate the topology information as a prior in the Bayesian approach which controls the size of the selected model. This method is assessed by applying to DREAM3 and DREAM4 datasets and two real biological datasets.

## 2. Method

### 2.1. The Nonlinear Regression Model for GRN Inference

Consider an *G* × *T* matrix *Y*, where *T* is the number of the gene expression levels measured times and *G* is the number of genes. A DBN model represents probabilistic relationships between genes via a directed acyclic graph *ϑ*. In this graph, genes are represented by a set of nodes *V* = {*V*_1_,…, *V*_*G*_} and the interactions between genes are represented by a set of directed edges *E*⊆{(*i*, *j*) : *i*, *j* ∈ *V*}. A directed edge from node *i* to node *j* means gene *i* is a regulator of gene *j*. The probability distribution of genes *Y*_*t*_ given its parents can be expressed as

(1) $$\begin{array}{c} p\left(\;Y_{t}\;|\;Y_{t-1}\;\right)=\overset{G}{\underset{g=1}{\prod }}p\left(\;Y_{g,t}\;|\;Pa\left(\;Y_{g,t}\;\right)\;\right), \end{array}$$

where *Y*_*g*,*t*_ is the gene *g* expression level at time *t* and *Pa*(*Y*_*g*,*t*_) is the set of all the parent nodes of gene *g* at time *t*. In the case of the regression-based DBN, the conditional distribution *p*(*Y*_*g*,*t*_∣*Pa*(*Y*_*g*,*t*_)) can be written as

(2) $$\begin{array}{c} y_{g,t}=f^{t}\left(\;y_{-g,t-1}\;\right)+\varepsilon_{g},\;g=1,\ldots ,G,\;\;t=1,\ldots ,T, \end{array}$$

where *y*_*g*,*t*_ is the expression level of gene *g* and *y*_−*g*,*t*−1_ is the vector without *y*_*t*−1_:

(3) $$\begin{array}{c} y_{-g,t-1}=\left(\;y_{1,t-1},\ldots ,y_{g-1,t-1},y_{g+1,t-1},\ldots ,y_{G,t-1}\;\right). \end{array}$$

We assume that the GRN is a time-invariant network; thus *f*^*t*^(·) = *f*(·) and the error term *ε*_*g*_ ~ *N*(0, *σ*^2^). Although *f*(·) can be characterized by any nonlinear functional representation, [15] suggested using B-spline basis functions instead of using Fourier basis, wavelets, or other nonlinear basis functions because of the pattern of the relationship between genes is unknown. Therefore, we also use B-spline basis functions in this article and the regulatory relationships can be written as

(4) yg,t=μg+fy1,t−1+⋯+fyg−1,t−1+fyg+1,t−1+⋯+fyG,t−1+εg,

where *μ*_*g*_ is the intercept and *f*(*y*_*i*,*t*_) = ∑_*k*=1_^*M*^*β*_*ik*_*B*_*ik*_(*y*_*i*,*t*_). {*B*_*ik*_(*y*_*i*,*t*_)} are *M* B-spline basis functions of degree *l* and *β*_*ik*_ is the parameters to estimate from data. Let {*κ*_*i*_} be the set of *r* equally spaced knots with min⁡{*y*_*i*_} = *κ*_*i*1_ < *κ*_*i*2_ < ⋯<*κ*_*ir*_ = max⁡{*y*_*i*_}, and *M* = *r* + *l*. We get rid of the subscript *g* for the variables for simplicity of notation. Then the regression equation can be written as

(5) $$\begin{array}{c} y=\mu +X\beta +\varepsilon , \end{array}$$

where *X* is the bases matrix of size *T* × *MG* and *β* is the corresponding coefficients vector.

### 2.2. Incorporating the Topology Information and Bayesian Inference

We use the Bayesian group lasso method proposed in [22]; the hierarchical Bayesian model is

(6) Y ∣ X,β,σ2~NnXβ,σ2In,βg ∣ σ2,τg2~γNmg0,σ2τg2Img+1−γgδ0βg,g=1,2,…,G,τg2~Gammamg+12,λ22,g=1,2,…,G,σ2~Inverse  Gammaa,b,γg~Bernoullip,p~Betac,d,

where *m*_*j*_ = 1 for *j* = 1 and *m*_*j*_ = *M* otherwise. Here we use a spike and slab prior on *β* and get the ranking of the potential regulatory links from *γ*. Although we can place a positive and very small *p* as a prior when the in-degree of the target gene is small, there are still a lot of false positive interactions to be predicted. Inspired by the idea of maxP technique proposed by [20], we use a prior proposed in [23], to place a restriction on *γ*, that only allow the model to be of small size.

(7) γ~Bernoullipγ≤k,γ=0otherwise.

Here the integer-valued hyperparameter *k* restricts the maximum number of parents for the target gene in each iteration. However, there are still some genes regulated by a large number of genes. Therefore, a fixed *k* will affect the accuracy of the prediction. Thus a uniform prior on [1, *m*] is placed on *k*, where *m* is a predetermined integer. Then the model becomes

(8) Y ∣ X,β,σ2~NnXβ,σ2In,βg ∣ σ2,τg2~γNmg0,σ2τg2Img+1−γgδ0βg,g=1,2,…,G,τg2~Gammamg+12,λ22,g=1,2,…,G,σ2~Inverse  Gammaa,bpγ=γ~Bernoullip,γ≤k,γ=0,otherwise,p~Betac,d,k~uniform1,m.

The likelihood is

(9) py ∣ X,β,σ2∝σ2−n/2exp⁡−y−XβTy−Xβ2σ2.

According to the prior and the likelihood above, the joint posterior distribution on data is

(10) pβ,σ2,τ2,γ ∣ Y,X∝σ2−n/2·exp⁡−y−XβTy−Xβ2σ2·∏g=1Gγσ2τg2−mg/2exp−βgTβg2σ2τg2+1−γδ0βg∏g=1Gλ2mg+1/2τg2mg+1/2−1·exp−λ22τg2σ2−a−1exp−bσ2pc−11−pd−1pγpk.

The Gibbs sampling scheme is as follows: We use *β*_−*g*_ = (*β*_1_,…, *β*_*g*−1_, *β*_*g*+1_,…, *β*_*G*_) to denote the coefficient vector *β* without the *g*th group and *X*_−*g*_ = (*X*_1_,…, *X*_*g*−1_, *X*_*g*+1_,…, *X*_*G*_) to denote the covariate matrix corresponding to *β*_−*g*_. The full conditions of (*γ*_*g*_ = 1, *β*_*g*_) and (*γ*_*g*_ = 0, *β*_*g*_) are

(11) pγg=1,βg ∣ rest∝2πτg2σ2−mg/2·exp⁡−βgTΣgβg−2ugTβg2σ2pγg=1,pγg=0,βg ∣ rest∝exp⁡−βgTXgTXgβg−2ugTβg2σ2pγg=0·δ0βg,

where *μ*_*g*_ = Σ_*g*_*X*_*g*_^*T*^(*Y* − *X*_−*g*_*β*_−*g*_) and Σ_*g*_ = (*X*_*g*_^*T*^*X*_*g*_ + (1/*τ*_*g*_^2^)*I*_*m*_*g*__)^−1^.

Integrating out *β*_*g*_, we have

(12) pγg=1,βg ∣ rest∝pγg=1τg2−mg/2Σg1/2·exp⁡XgTY−X−gβ−gTΣgXgTY−X−gβ−g2σ2,pγg=0,βg ∣ rest∝pγg=0.

From these equations, we can draw *γ*_*g*_ through

(13) $$\begin{array}{c} p\left(\;\gamma_{g}=0\;|\;rest\;\right)=\frac{p}{p+\left(1-p\right){\left(\tau_{g}^{2}\right)}^{-m_{g}/2}{\left|\Sigma_{g}\right|}^{1/2}\exp\left({\left(X_{g}^{T}\left(y-X_{-g}\beta_{-g}\right)\right)}^{T}\Sigma_{g}\left(X_{g}^{T}\left(y-X_{-g}\beta_{-g}\right)\right)/2\sigma^{2}\right)}. \end{array}$$

Then the full conditional posterior distribution of *β*_*g*_ is

(14) $$\begin{array}{c} p\left(\;\beta_{g}\;|\;\gamma_{g}=1,rest\;\right)\propto \exp\left(\;-\frac{\beta_{g}^{2}-2\mu_{g}}{2\sigma^{2}\Sigma_{g}}\;\right). \end{array}$$

Thus, the full conditional distribution of *β*_*g*_ is a normal distribution:

(15) βg ∣ γg=1,rest~Nug,σ2Σg,pβg=0 ∣ γg=0,rest=1.

The full conditions of (*τ*_*g*_^2^, *γ*_*g*_ = 1) and (*τ*_*g*_^2^, *γ*_*g*_ = 0) are

(16) pτg2,γg=1∝τg2−1/2exp⁡−βgTβg2σ2τg2−λ22τg2,pτg2,γg=0∝τg2mg+1/2−1exp⁡−λ22τg2.

Then the full conditional distribution of *τ*_*g*_^2^ is

(17) 1τg2,γg=1 ∣ rest~Inverse  Gaussianλσβg2,λ2,1τg2,γg=0 ∣ rest~Inverse  Gammamj+12,λ22.

The full conditional distribution of *σ*^2^ is

(18) pσ2 ∣ rest∝σ2−n/2−1/2∑g=1GmgZg−a−1·exp⁡−y−XβTy−Xβ+βTDτ−1β+b2σ2.

Then the conditional posterior distribution of *σ*^2^ is

(19) σ2 ∣ rest~Inverse  Gamman2+12∑g=1GmgZg+a,12Y−XβTY−Xβ+βTDτ−1β+b,

where *Z*_*g*_ = {1, if  *γ*_*g*_ = 0; 0, if  *γ*_*g*_ ≠ 0} and *D*_*τ*_ = diag⁡{*τ*_1_^2^, *τ*_2_^2^,…, *τ*_*G*_^2^}. And it can be verified that the conditional posterior distributions of other parameters are

(20) p ∣ rest~Betac+∑g=1GZg,b+∑g=1Gmg−∑g=1GZg,k ∣ rest~uniform∑g=1GZg,m.

And a Monte Carlo EM algorithm is used to estimate *λ*:

(21) $$\begin{array}{c} \lambda^{\left(k\right)}=\sqrt{\frac{p+G}{\sum_{g=1}^{G}E_{\lambda^{\left(k-1\right)}}\left[\tau_{g}^{2}\;|\;Y\right]}}, \end{array}$$

where *p* equal to 1 + *m*_*j*_ × (*G* − 1) is the number of the total regressors and *E*_*λ*^(*k*−1)^_[*τ*_*g*_^2^∣*Y*] can be replaced by the sample average of *τ*_*g*_^2^ generated in the *k* − 1th step of the Gibbs sampler. We choose the second half of the samples and the result is the average of the samples.

## 3. Results

To demonstrate the effectiveness of the topology information and the B-spline functions, our method is used to infer GRNs from in silico time-series data and real biological data; a linear model with topology information and a nonlinear model without topology information are also applied as competing methods. Here we use the time-series data in DREAM3 and DREAM4 challenges as the in silico data, and we use a cell cycle regulatory subnetwork in* Saccharomyces cerevisiae* and Human Hela cell network as the real biological datasets. We generate 10000 samples from the posterior distribution and choose the second half of the samples to derive the results. The posterior estimates of all the parameters are obtained through the posterior averages of the chains. For the B-spline functions, we adopt the setting as [14] and use a cubic B-spline with 10 interior knots. Here we choose *m* = 5 in our experiments.

### 3.1. Application to In Silico Networks

We first evaluate our method on DREAM4 challenges networks of sizes 10 and 100 [24–26]. The size 10 network data consists of 5 simulated networks, each of which consists of 21 time points and 5 replicates. The size 100 network data also consists of 5 simulated networks, each of which consists of 21 time points and 10 replicates. We also evaluate our method on DREAM3 challenges networks of sizes 10, which is also used in [27]. This data consists of 5 simulated networks, each of which consists of 21 time points. There are also steady-state data provided by the DREAM4 challenge. However, we only focus on time-series data in this article. Although the winning entry in DREAM4 competition used only the knock-out data [28] and combining the time-series and steady-state data can achieve much better results [27, 29], it is infeasible to do knock-out experiments for all genes in practice and generally the knock-out experiments only are done for a small part of genes [30].

Each of the five networks is inferred using all available time-series data, and the area under the receiver operating characteristic (AUROC) curve and the area under precision-recall (AUPR) curve are computed according to the gold standard network topology provided by DREAM3 and DREAM4 challenge. The prediction performances on the DREAM4 10-gene networks and 100-gene networks are summarized in Tables 1 and 2.  Table 1 shows that the Bayesian lasso and Bayesian group lasso perform similarly on size 10 data while the BGL_prior has a better performance than the methods above in both average AUROC and AUPR. For net 2 and net 5, the BGL_prior outperforms other methods significantly. We also compared our method with another 2 dynamic Bayesian network methods [31]; the result of our method is also comparable to these methods.  Table 2 shows that the nonlinear model performs poorly on this dataset; while the topology information can remarkably improve the prediction performance of the nonlinear model, the Bayesian group lasso with topology information outperforms the Bayesian group lasso methods in both AUROC and AUPR, and these methods also have higher AUROC than linear model, although the AUPR is a little worse. Compared with the results of the other 2 DBN methods, the result of the Bayesian lasso is similar to them and our method still has the highest average AUROC. The prediction performances on the DREAM3 10 gene networks are summarized in Table 3. We also compared our method with another additive model based on ODE [27] and Inferelator 1.0 [32]. For Ecoli 1, Ecoli 2, Yeast 2, and Yeast 3, the 3 additive models perform better than the linear model; for Yeast 1, although BL performs much better than BGL, the BGL_prior still gets slightly better results. The average results show that BGL_prior outperforms the other methods in both AUROC and AUPR.

### 3.2. Application to IRMA Network

The IRMA network data is a subnetwork embedded in* Saccharomyces cerevisiae* consisting of 5 genes: CBF1, GAL4, SWI5, GAL80, and ASH1. Both of the two time-series gene expressions include switch-on data and switch-off data. The switch-on data is taken from 5 experiments and the switch-off data is taken from 4 experiments with a total of 142 samples measured by [33] and also used in [20, 34]. The IRMA network is well studied and is a gold standard network. This network also has a fixed topology and the genes in the network are not regulated by other yeast genes. Here we use the precision rate (PR = TP/(TP + FP)), recall rate (RR = TP/(TP + FN)), and *F*-measure = (2 · PR · RR)/(PR + RR) to evaluate the performance and select a best threshold as [35]. The signs of the interactions and self-regulations are not considered; thus the total number of the potential interactions is 20.  Table 4 shows the inference performance for the IRMA network. The nonlinear model still performs better than linear model. The method with the prior has a higher TP than the Bayesian group lasso, which implies that the topology information improves the performance. Comparing with another B-spline based method [14] and the method used and compared in [36], although our method cannot achieve the best performance, it is still comparable to the TSNIF and performs much better than another B-spline based method.

### 3.3. Application to Hela Network

We then apply our method on the cell cycle genes in human cancer cell lines (HeLa) which were analyzed by Whitfield [37]. A subnet consisting of 9 Hela cell genes was extracted by Sambo et al. [38] and the topology of this gene regulatory network is determined in the BioGRID database. They also developed a method called CNET to analysis the Hela network. This network is also analyzed by Lozano et al. [39] and Shojaie and Michailidis [40]; they proposed 2 *l*_1_ penalized method, grpLasso, and TAlasso to infer causal interactions.

Here we use the third experiment of Whitfield [37] as the previous studies, consisting of 47 samples. The results of CNET, grpLasso, and TAlasso are taken from [40].  Table 5 shows the inference performance for the Hela network. Comparing with the BGL, the BGL_prior has a higher precision. Comparing with other methods, the penalized method seems to perform better than another B-spline based method and has a similar performance to the other 2 *l*_1_ penalized methods and all the true positives of Morrissey's method are also found by BGL and BGL_prior. On the other hand, the interactions from RFC4 to CDC2 and CDC2 to CCNE1 are found not only by BGL and BGL_prior, but also by 2 of other 3 comparable methods. It may be because these interactions exist in real regulatory network but are not included in the BioGRID dataset.

## 4. Conclusion

In this study, we propose a fully Bayesian method, based on B-spline, group lasso, and topology information to infer gene regulatory network from time-series data. We use B-spline functions to capture the nonlinear interactions between genes, *l*_1_ norm penalty to prevent overfitting, and topology information, the knowledge of the exponential decrease in in-degree that most genes have only a small number of regulators as a prior. A spike and slab prior is used to facilitate variable selection by putting a multivariate point mass at 0_*m*×1_ for an *m*-dimensional coefficients group. The performance of the proposed method is demonstrated by applications to the DREAM4 in silico data of sizes 10 and 100 network challenges and the real biological data of IRMA and Hela cell network. The results show that the topology information indeed contributes to the gene regulatory network inference which can improve the AUROC remarkably of the DREAM4 in silico data and improve the results of the IRMA network and Hela cell data. B-spline regression model also performs better than linear model in real biological data. Therefore, our method is an effective way of inferencing gene regulatory network from the time-series data.

## Figures and Tables

**Table 1:** The prediction performances on the DREAM4 10-gene networks.

	Method	Net 1	Net 2	Net 3	Net 4	Net 5	Average
AUROC	BL	0.7956	0.6334	0.6356	0.8292	0.8034	0.7394
	BGL	0.7956	0.6537	0.6507	0.8422	0.8194	0.7523
	BGL_prior	0.8267	0.7188	0.6640	0.8472	0.8547	0.7823
	G1DBN	0.73	0.64	0.68	0.85	0.92	0.7640
	VBSSM	0.73	0.66	0.77	0.80	0.84	0.7600

AUPR	BL	0.4222	0.3711	0.3926	0.5235	0.4683	0.4355
	BGL	0.4613	0.3582	0.3781	0.5392	0.3368	0.4147
	BGL_prior	0.4750	0.4090	0.3062	0.6207	0.5022	0.4626
	G1DBN	0.37	0.34	0.45	0.69	0.77	0.5240
	VBSSM	0.38	0.41	0.49	0.46	0.64	0.4760

**Table 2:** The prediction performances on the DREAM4 100-gene networks.

	Method	Net 1	Net 2	Net 3	Net 4	Net 5	Average
AUROC	BL	0.7180	0.6040	0.6748	0.6551	0.7269	0.6758
	BGL	0.5768	0.5915	0.6148	0.5692	0.5829	0.5878
	BGL_prior	0.7117	0.6745	0.7062	0.6859	0.7327	0.7029
	G1DBN	0.68	0.64	0.68	0.66	0.72	0.6760
	VBSSM	0.59	0.56	0.59	0.67	0.71	0.6240

AUPR	BL	0.1177	0.0830	0.1154	0.1103	0.0776	0.1008
	BGL	0.0318	0.0984	0.0440	0.0685	0.0409	0.0567
	BGL_prior	0.508	0.0656	0.0967	0.1021	0.0730	0.0776
	G1DBN	0.11	0.10	0.13	0.10	0.11	0.1100
	VBSSM	0.08	0.05	0.11	0.10	0.09	0.0860

**Table 3:** The prediction performances on the DREAM3 10-gene networks.

	Method	Ecoli 1	Ecoli 2	Yeast 1	Yeast 2	Yeast 3	Average
AUROC	BL	0.4948	0.6880	0.6200	0.4412	0.4091	0.5306
	BGL	0.5339	0.7813	0.5525	0.5348	0.4646	0.5734
	BGL_prior	0.6237	0.7876	0.6363	0.5034	0.4987	0.6099
	Inferelator 1.0	0.49	0.52	0.56	0.45	0.48	0.5000
	Additive ODE	0.53	0.54	0.45	0.53	0.48	0.5060

AUPR	BL	0.2455	0.5325	0.2981	0.2979	0.2009	0.3150
	BGL	0.2076	0.6096	0.2749	0.2871	0.2199	0.3220
	BGL_prior	0.2427	0.6144	0.2795	0.2544	0.2347	0.3251
	Inferelator 1.0	0.15	0.21	0.22	0.33	0.28	0.2380
	Additive ODE	0.16	0.20	0.10	0.31	0.23	0.2000

**Table 4:** The prediction performances on IRMA network.

Method	TP	FP	TN	FN	PR	RR	*F*
BL	5	9	3	3	0.3571	0.6250	0.4545
BGL	4	3	9	4	0.5714	0.5000	0.5333
BGL_prior	5	3	9	3	0.6250	0.6250	0.6250
Morrissey's method	1	1	13	5	0.5	0.1667	0.2500
TSNIF	5	2	10	3	0.7143	0.6250	0.6667
BANJO	5	6	6	3	0.4545	0.6250	0.5263

**Table 5:** The prediction performances on Hela network.

Method	TP	FP	TN	FN	PR	RR	*F*
BL	2	9	54	7	0.1818	0.2222	0.2000
BGL	5	18	45	4	0.2174	0.5556	0.3125
BGL_prior	5	14	49	4	0.2632	0.5556	0.3571
Morrissey's method	3	15	48	6	0.1667	0.6667	0.2222
TALasso	3	7	56	6	0.3	0.3333	0.3158
grpLasso	4	13	50	5	0.2353	0.4444	0.3076
CNET	4	7	56	5	0.3636	0.4444	0.4000
